# Shifts in the Oral Microbiota During a Four-Week Commercial Saturation Dive to 200 Meters

**DOI:** 10.3389/fphys.2021.669355

**Published:** 2021-04-27

**Authors:** Roxane Monnoyer, Kjersti Haugum, Jacky Lautridou, Arnar Flatberg, Astrid Hjelde, Ingrid Eftedal

**Affiliations:** ^1^Department of Circulation and Medical Imaging, Faculty of Medicine and Health Sciences, NTNU Norwegian University of Science and Technology, Trondheim, Norway; ^2^Department of Clinical and Molecular Medicine, Faculty of Medicine and Health Sciences, NTNU Norwegian University of Science and Technology, Trondheim, Norway; ^3^Department of Medical Microbiology, Clinic of Laboratory Medicine, St. Olavs Hospital, Trondheim University Hospital, Trondheim, Norway; ^4^Faculty of Nursing and Health Sciences, Nord University, Bodø, Norway

**Keywords:** acclimatization, decompression, heliox saturation, microbiome, metagenomic, bacterial phyla, bacterial genera

## Abstract

During commercial saturation diving, divers live and work under hyperbaric and hyperoxic conditions. The myriads of bacteria that live in and on the human body must adjust to the resultant hyperbaric stress. In this study, we examined the shifts in bacterial content in the oral cavity of saturation divers, using a metagenomic approach to determine the diversity in the composition of bacterial phyla and genera in saliva from 23 male divers before, during, and immediately after 4 weeks of commercial heliox saturation diving to a working depth of circa 200 m. We found that the bacterial diversity fell during saturation, and there was a change in bacterial composition; with a decrease at the phylum level of obligate anaerobe *Fusobacteria*, and an increase of the relative abundance of *Actinobacteria* and *Proteobacteria*. At the genus level, *Fusobacterium*, *Leptotrichia*, *Oribacterium*, and *Veillonella* decreased, whereas *Neisseria* and *Rothia* increased. However, at the end of the decompression, both the diversity and composition of the microbiota returned to pre-dive values. The results indicate that the hyperoxic conditions during saturation may suppress the activity of anaerobes, leaving a niche for other bacteria to fill. The transient nature of the change could imply that hyperbaric heliox saturation has no lasting effect on the oral microbiota, but it is unknown whether or how a shift in oral bacterial diversity and abundance during saturation might impact the divers’ health or well-being.

## Introduction

Commercial saturation diving is used to perform long-term subsea work at greater depths. During saturation diving operations, the divers live within a pressurized, hyperbaric chamber system in a heliox atmosphere (a mix of oxygen and helium) for longer periods, normally limited to 28 days (DMAC, 2006). The divers commute to work from the hyperbaric chamber system to the sea bottom in a diving bell. Although saturation diving is generally considered safe, the environmental conditions encountered by the divers are still a matter of health concern (Brubakk et al., 2014). Preserving health in harsh environments requires successful acclimation of the body’s physiological mechanisms. In this respect, the multiple microorganisms living in or on the body are also involved; i.e., the various microbiota that constitutes the human microbiome. Since the first appearance of the term microbiota in 2001 (Lederberg and McCray, 2001) and the recent development of metagenomics study tools, a large number of studies have emerged emphasizing the dual role of microorganisms inhabiting the human body in health protection as well as the development of diseases (Scannapieco, 2013; Wade, 2013; Yamashita and Takeshita, 2017; Sharma et al., 2018; Zheng et al., 2020). They interact with the host’s immune system and affect central metabolic processes (Chu and Mazmanian, 2013; Belkaid and Hand, 2014).

Divers are exposed to several stress factors during hyperbaric saturation (Bosco et al., 2018), which may also affect the composition and activity of the microbial communities. Bacteria residing in the divers’ oral cavity, the oral microbiota, come directly into contact with the hyperbaric breathing gases. It has been proposed that oral bacteria contribute to their host’s health and fitness beyond the oral cavity. For instance, they may be involved in the control of cardiovascular function by nitric oxide (NO) *via* their essential function in the regulation of nitrate (NO_3_^−^) production (Hyde et al., 2014; Cutler et al., 2019), and are thought to play a role in autoimmune disease susceptibility (Nikitakis et al., 2017). The effects of hyperbaric heliox saturation on the human oral microbiome have yet to be determined.

This study was designed to examine the effects of commercial saturation diving on the bacterial content of the oral microbiota. A metagenomic approach was used to determine the composition of bacterial phyla and genera in divers’ saliva before, during, and immediately after 4 weeks of commercial heliox saturation diving.

## Materials and Methods

### Ethics

The study was conducted during a commercial saturation diving operation in the Mediterranean Sea, March–April 2018. The protocol was approved in advance by the Norwegian Regional Committee for Medical and Health Research Ethics (REK), approval number 2018/1184. Divers who passed the pre-saturation medical examination and were committed to saturation onboard the Dive Support Vessel (DSV) Deep Arctic were eligible for participation. They were informed of the aim and scope of the study and provided written consent before inclusion. The experimental procedures were conducted according to the Declaration of Helsinki principles for ethical human experimentation.

### Study Subjects

Initially, 30 certified saturation divers, all male non-smokers, were enrolled in the study for the duration of a 28-day work assignment in hyperbaric heliox saturation. All held valid health certificates for work in saturation and fulfilled the operator’s requirement for aerobic fitness with maximum oxygen uptake capacity VO_2max_ ≥ 40 L/min. Table 1 describes the study subject characteristics prior to saturation. The study did not interfere with the divers’ lifestyle, whether diet, activity outside of operational requirements, nor the use of dentifrice. In the final analysis, we included only divers from whom four sets of saliva samples were successfully obtained, resulting in data from 23 of the 30 divers.

**Table 1:** Study subject characteristics prior to saturation (*n* = 23).

	Mean	Range
Age (years)	44	31–60
BMI (kg/m^2^)	26.8	20.2–31.2
VO_2_ max (L/min)	48	44–60

BMI, body mass index; VO_2_ max, maximum oxygen uptake capacity.

### Saturation Diving

Saturation diving was performed according to the contractor’s procedures as previously described (Łuczyński et al., 2019). In summary, the divers were compressed in a heliox atmosphere over a period of about 6 h to a storage depth of 178–192 meters of seawater (msw). They remained at storage depth throughout the bottom phase for 21 days, during which time they performed daily shifts of underwater work in teams of three at depths of 191–207 msw. Each shift lasts 12 h, 7 days per week. A dive bell was used to transport the divers between the pressure chamber and the underwater work site. When the bottom phase was completed, the divers were decompressed back to atmospheric pressure over a period of 8 days.

**Figure 1:**
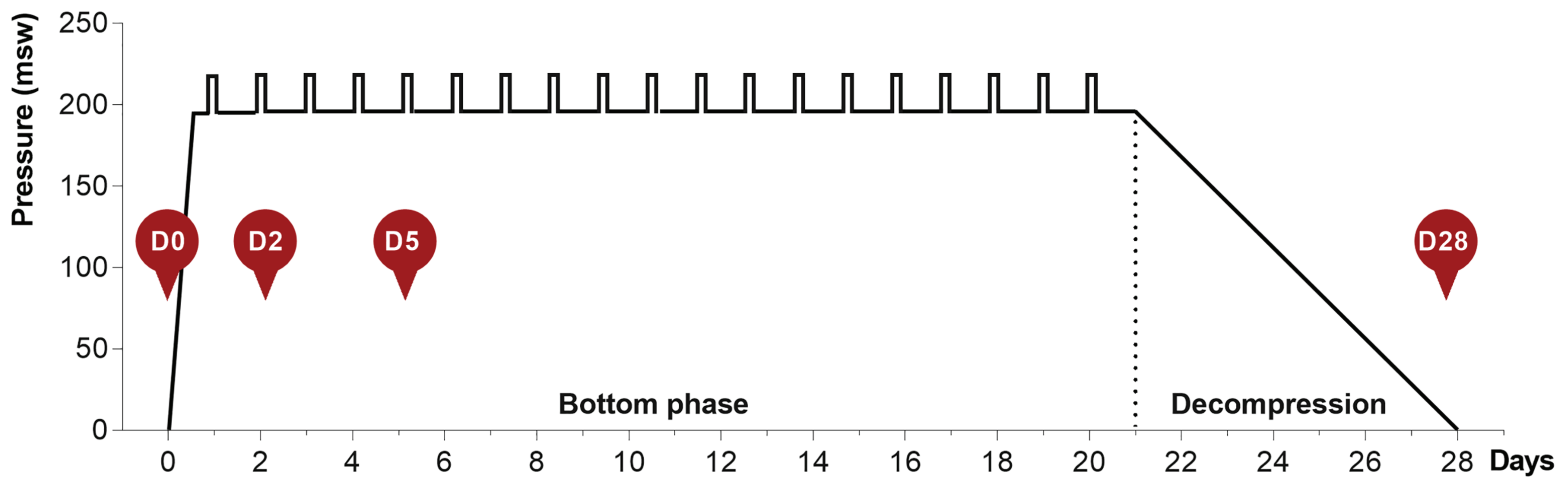
The 28-day heliox saturation profile. Hyperbaric saturation was done in a heliox atmosphere with 6 h compression, 21-day bottom phase, and 7-day decompression. Daily underwater work excursions (bell-runs) in the bottom phase are indicated by vertical bars. Saliva collection time-points are shown as red circles: D0, before saturation; D2 and D5, days 2 and 5 of the bottom phase; D28, after decompression.

During the bottom phase, the partial pressure of oxygen (ppO_2_) was kept at 40 kPa in the pressure chamber and 60–80 kPa during the bell-runs. During decompression, the ppO_2_ was increased to 50 kPa until a depth of 13 msw was reached, and from there on ppO_2_ was gradually reduced to 21%. After reaching the surface, the divers stayed on the vessel for another 24 h for observation for decompression sickness, before they left the vessel. The saturation profile is shown in Figure 1.

### Saliva Collection

Saliva samples were collected at four time-points for each diver, as shown in Figure 1. D0 is at surface before saturation, D2 and D5 are on days 2 and 5 during bottom phase, and D28 is the day decompression was completed and the divers were back to surface. The divers themselves collected the saliva and were instructed not to brush their teeth before sampling. All sampling was undertaken ≥2 h after the last meal and collected in an all-in-one kit for stabilization of microbial nucleic acids (OMNIgene ORAL OM-505, DNA Genotek, Ottawa, Canada). Samples obtained on days 2 and 5, while the divers were pressurized, were decompressed back to surface pressure through an airlock shortly after collection. During the collection period, samples were stored in a fridge in the vessel hospital. At the end of the operation, all samples were transported collectively to the Norwegian University of Science and Technology (NTNU) for analysis. The bottom phase time-points for sample collection were chosen based on earlier reports on hematology in saturation diving, and are in agreement with recent data on mice lung and gut microbiota in response to hyperoxia (Hofso et al., 2005; Ashley et al., 2020). At the time of sample collection, the divers had already performed at least on round of heliox saturation during the same diving campaign. The baseline (day 0) samples, therefore, also represent the status of the diver’s microbiota 4–6 weeks after a similar exposure.

### 16S Library Preparation for Metagenome Sequencing

16S metagenomic sequencing libraries were prepared according to the QIAseq 16S Region Panel protocol (Qiagen, Hilden, Germany). In brief, 4 nanograms (ng) of genomic DNA extracted from the saliva samples (*n* = 92) were used as a template for PCR of the 16S V2–V3 and V4–V5 regions, in separate reactions using Illumina primers/reagents. The resulting 16S amplicons were purified using AMPure XP beads (Beckman Coulter, Inc., Indianapolis, IN, United States). Purified PCR products from each sample were pooled and thereafter subjected to a second PCR amplification step, where dual samples indices and Illumina sequencing adaptors were added, according to the manufacturer’s instructions. A second PCR clean-up step was performed using AMPure XP beads (Beckman Coulter, Inc., Indianapolis, IN, United States), before the validation of the sequencing library using an Agilent High Sensitivity DNA Kit on a BioAnalyzer (Agilent Technologies, Santa Clara, CA, United States). Individual sample libraries were pooled and normalized to 10 pM, prior to sequencing of 2 × 300 cycles with a MiSeq V3 flowcell on a MiSeq instrument, according to the manufacturer’s instructions (Illumina, Inc. San Diego, CA, United States). Sequence reads were demultiplexed and converted from BCL to fastq file format using bcl2fastq2 conversion software V2.20.0422 (Illumina, Inc. San Diego, CA, United States).

### Bioinformatics

The sequencing data were merged and analyzed using the Quantitative Insights Into Microbial Ecology (QIIME2, version 2019.10) pipeline (Caporaso et al., 2010). All following procedures in this section were conducted in the QIIME2 environment using QIIME2 plugins. Demultiplexed paired-end reads from MiSeq (2 × 300 bp) were trimmed to remove primers and poor quality bases with fastp (version 0.20.0; Chen et al., 2018). The trimmed sequences were denoised and clustered with DADA2 (Callahan et al., 2016). The generated amplicon sequence variants (ASVs) were assigned to taxonomy using a targeted classifier. Briefly, we extracted sequences from the SILVA database (version 132) at a similarity threshold of 99% with locus-specific sequences from V2–V3 and V4–V5 QIAseq 16S primers and a targeted Naive Bayes trained on the extracted sequences. The QIIME2 phylogeny plugin was applied to construct the rooted phylogenetic tree by employing the FastTree program (Price et al., 2009). Based on the taxonomy generated, we filtered our feature-table to include only assigned reads of the taxonomic kingdom Bacteria and exclude reads assigned to mitochondria or chloroplasts. The generated BIOM file and phylogenetic trees were further imported into Phyloseq for comparison and visualization (McMurdie and Holmes, 2013).

### Statistical Analysis

To estimate the diversity of the oral microbiota within individuals, i.e., the richness and evenness of the bacterial community (Kim et al., 2017), different indexes of the alpha diversity within samples such as the Shannon index were calculated by the function estimate_richness and the beta diversity between samples was calculated on a Bray-Curtis distance measure by the function ordinate using the Phyloseq package on R studio. Significance between groups in the PCoA subspace of the first two components was estimated by permuted Anova (permANOVA) from the vegan package. Statistical analysis on the Shannon index and the relative abundance at the phylum and genus level was done in IBM SPSS Statistics software Version 26.0. Normal distribution of the data was confirmed by visual inspection of Q-Q plots and Shapiro-Wilk’s test for normality (*p* > 0.05), either directly or after the data were transformed. If the data were still non-normal after transformation, a non-parametric analysis was applied. A one-way repeated measures ANOVA, with Bonferroni *post hoc* adjustment for multiple comparisons, was used to assess within diver differences in Shannon index and relative abundance of phyla and genera between the four time-points. Differences were considered significant at *p* < 0.05. If the assumption of sphericity was violated, as assessed by Mauchly’s test which assumes homogeneity for *p* > 0.05, a Greenhouse-Geisser correction was applied. In the case of non-normal data, Friedman’s test was applied and pairwise comparisons were performed with a Bonferroni correction for multiple comparisons.

### Data Repository

The data for this study have been deposited in the European Nucleotide Archive (ENA) at EMBL-EBI under accession number PRJEB40804.^1^

## Results

**Figure 2:**
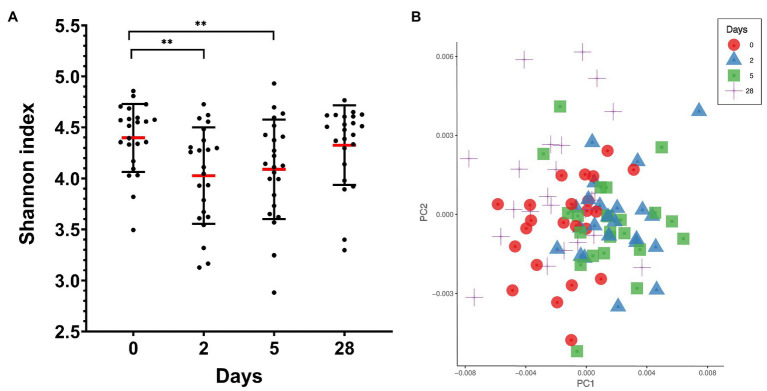
Oral microbiota diversity in saliva from commercial saturation divers (*n* = 23) before, during, and after 28 days of heliox saturation. **(A)** The alpha diversity within samples, i.e., species richness and evenness, is given by the Shannon index (^**^p < 0.001). Means (red line) and individual values are shown. Error bars are ±1 SD. **(B)** Principal coordinate analysis (PCoA) plot of beta-diversity for individual samples showing the first two principal components (PC1 and PC2) using Bray-Curtis distances (*p* = 0.001 using permANOVA). Colored symbols indicate times of sample collection. In both panels, day 0, before saturation; days 2 and 5, during the bottom phase; day 28, after decompression.

Ten different bacterial phyla were detected by taxonomic analysis of the 16S rRNA sequencing data from saliva collected from 23 divers before, during, and at the end of 4 weeks of commercial heliox saturation diving. The analysis of alpha diversity within samples, i.e. species richness and evenness using the Shannon index, showed a significant drop during the bottom phase (4.028 ± 0.47 for day 2, and 4.089 ± 0.48 for day 5, *p* < 0.001), compared to the baseline (day 0; 4.398 ± 0.33). However, the alpha diversity returned to baseline after the decompression (Figure 2A). The beta diversity is shown in Figure 2B.

**Figure 3:**
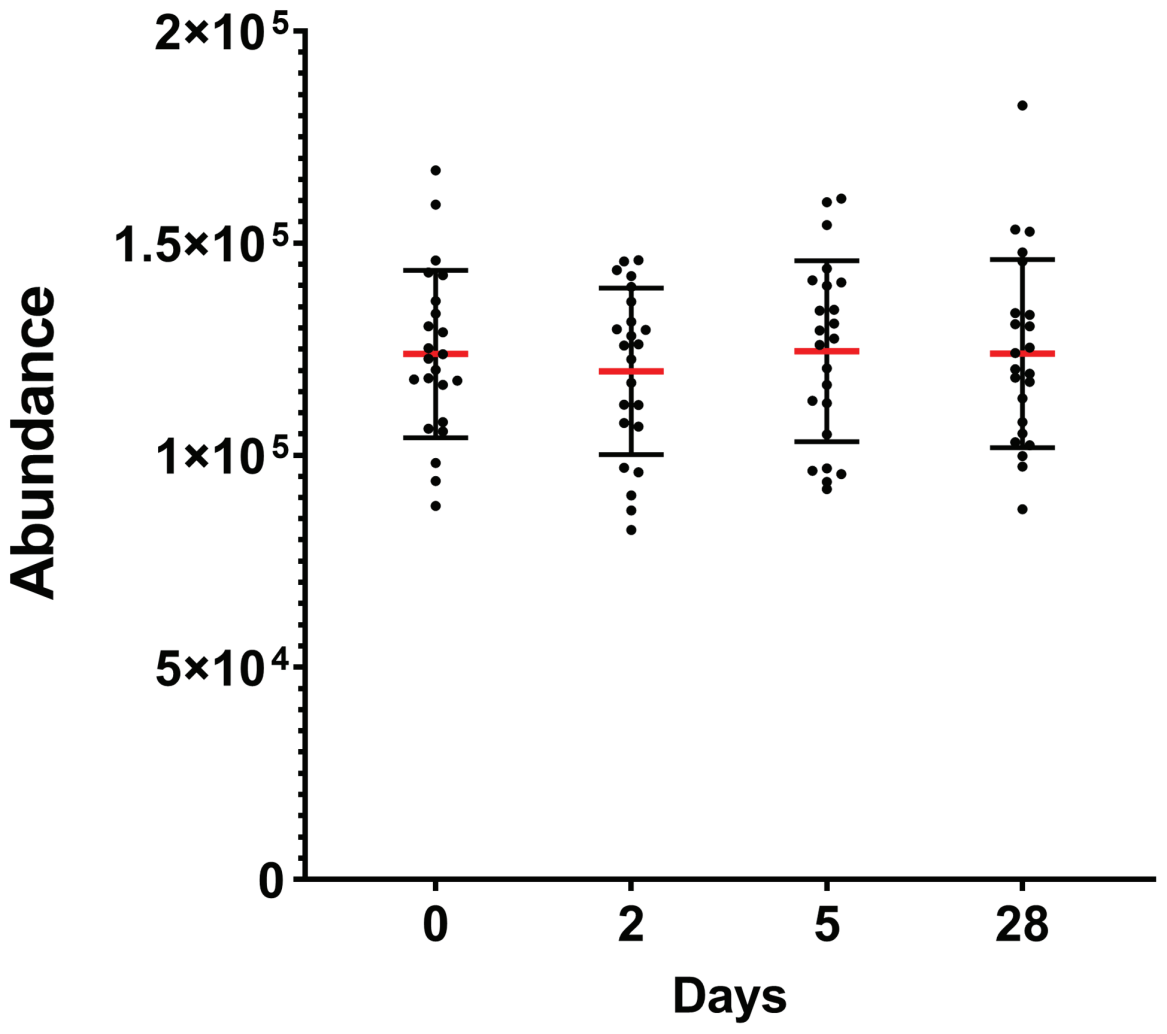
Total abundance of the oral bacterial communities in saliva from commercial saturation divers (*n* = 23) before, during, and after 28 days of heliox saturation, displayed as the number of reads for all detected OTU after 16S sequencing. For each time-point, means (red line) and individual values are shown. Error bars are ±1 SD. Day 0, before saturation; days 2 and 5, during the bottom phase; day 28, after decompression.

Taken altogether, the total abundance of the 10 detected phyla did not significantly change during heliox saturation (Figure 3).

**Figure 4:**
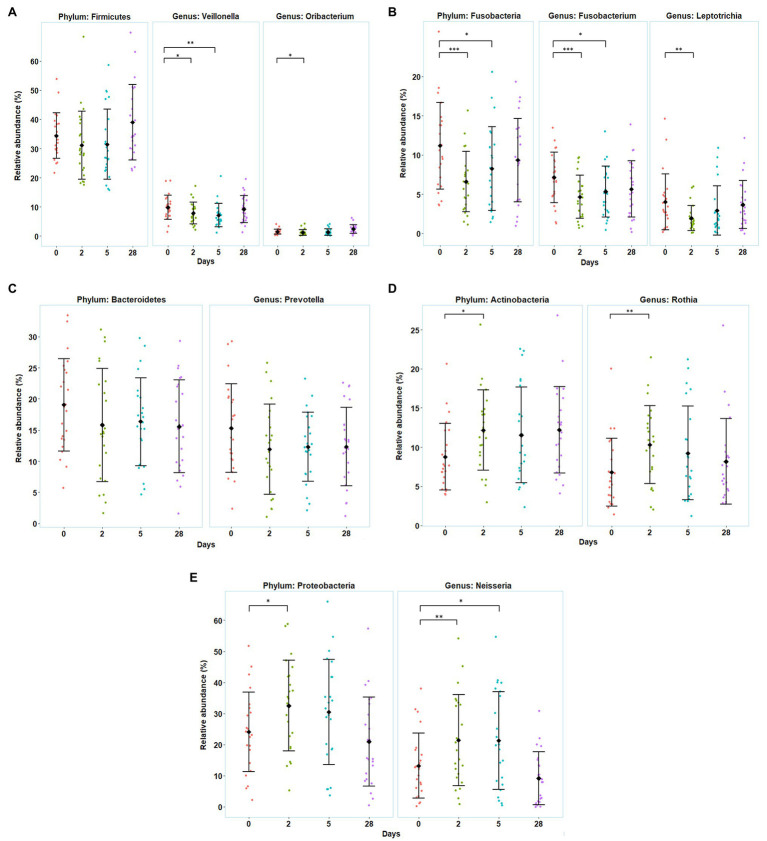
Variation in the divers’ oral microbiota before (day 0), during the bottom phase (days 2 and 5), and after decompression (day 28) from heliox saturation diving. **(A–E)** Observed shifts in the relative abundance of the five most abundant bacterial phyla, along with their significantly altered genera. For each time-point: mean (♦) and individual values are shown. Error bars are ±1 SD (^*^p ≤ 0.05; ^**^p ≤ 0.01; and ^***^p ≤ 0.001). The relative abundance is given in percentage of total bacterial sequences.

In general, five phyla were dominant in all the samples: *Firmicutes* (34%), *Proteobacteria* (27%), *Bacteroidetes* (17%), *Actinobacteria* (11%), and *Fusobacteria* (9%). Five other phyla were detected at lower levels (<1%), including *Patescibacteria*, *Epsilonbacteræota*, *Tenericutes*, *Spirochaetes*, and *Synergistetes*. Moving down the taxonomic tree, the most abundant significantly altered genera belonged to the phyla *Firmicutes* (genus *Veillonella* and *Oribacterium*), *Proteobacteria* (genus *Neisseria*), *Actinobacteria* (genus *Rothia* and *Actinomyces*), and *Fusobacteria* (genus *Fusobacterium* and *Leptotrichia*) (Figure 4).

The relative abundance of *Firmicutes*, *Fusobacteria*, and *Bacteroidetes* decreased during the first 5 days of the bottom phase, but only significantly so for *Fusobacteria*. Within the phylum *Firmicutes*, one of the dominant genera, *Veillonella*, decreased at day 2 (*p* = 0.047) and day 5 (*p* = 0.004), as well as the genus *Oribacterium*, that also decreased but only at day 2 of the bottom phase (*p* = 0.040; Figure 4A). In the phylum *Fusobacteria*, decreases in abundance were observed for the genus *Fusobacterium* at day 2 (*p* < 0.0005) and day 5 (*p* = 0.027), and in genus *Leptotrichia* at day 2 (*p* = 0.0022). We also observed a decrease in the genus *Prevotella* from *Bacteroidetes* but non-significant (Figures 4B,C). Conversely, during the bottom phase, at day 2, the abundance of *Actinobacteria* and *Proteobacteria* increased. In these phyla, genus *Rothia* (*Actinobacteria*) increased at day 2 (*p* = 0.009; Figure 4D), while genus *Neisseria* (*Proteobacteria*) increased at day 2 (*p* = 0.010) and day 5 (*p* = 0.022; Figure 4E). However, all changes observed during the bottom phase were abolished at the end of the dive.

## Discussion

In this study, we surveyed the oral bacterial microbiota of divers during 4 weeks of commercial heliox saturation diving. Using a taxonomic analysis through 16S rRNA sequencing, we identified 10 abundant bacterial phyla in saliva collected before, during, and immediately after saturation, and this composition is coherent with other studies that describe the human oral microbiota (Palmer Jr., 2014). We found that the microbial diversity was reduced during saturation and that the relative abundance of *Proteobacteria*, *Actinobacteria*, and *Fusobacteria* changed significantly. In each phylum, these changes were mostly due to their most abundant genera, including a shift from obligate anaerobes (*Fusobacterium*, *Leptotrichia*, *Veillonella*, and *Oribacterium*) to aerobic/facultative anaerobic bacteria (*Neisseria* and *Rothia*). To our knowledge, this is the first study to address the relationship between hyperbaric environments and oral microbiota.

In our study, we observed that the alpha diversity of the oral microbiota was reduced during hyperbaric heliox saturation, indicating that either the richness and/or evenness of the microbial communities changed. The relative abundance of *Fusobacteria* decreased during saturation, whereas *Proteobacteria* and *Actinobacteria* increased. The most probable explanation is that the evenness of the oral microbiota was impacted by the drop of *Fusobacteria* during the bottom phase together with the raise of *Proteobacteria* abundance, in particular the *Neisseria* genus – of which this phylum alone made up more than 30% of the total abundance during the bottom phase. Furthermore, the absence of change in the total abundance during saturation (Figure 2B) indicates that the environmental conditions inherent to the dive merely created a shift in the microbial balance between the different genera and therefore phyla. In apparent contrast with our results, a recent study on commercial saturation divers’ gut microbiota reported no change in alpha diversity, but a decreased abundance of the genus *Bifidobacterium*, which are in fact *Actinobacteria* (Yuan et al., 2019).

This discrepancy may be explained by the fact that the oral microbiome is directly in contact with the breathing mixture during the dive and thus is more reactive to the steep changes in pressure and oxygen availability. Oral bacteria can be classified according to their oxygen needs and their ability to metabolize it in different environments regarding each site of the mouth from tooth surfaces to supragingival and subgingival regions. Obligate aerobes are found to grow optimally at atmospheric concentrations of oxygen (~20%), whereas microaerophiles prosper best well-below normal atmospheric concentrations. Unlike obligate anaerobes that cannot tolerate oxygen and thrive only under anoxic conditions; facultative anaerobes can grow by fermentation, use other terminal electron acceptors for anaerobic respiration, or their ability to breathe aerobically. Aerotolerant anaerobes as the name suggests can tolerate the presence of oxygen, but do not benefit from aerobic respiration and thrive optimally without oxygen (Morris and Schmidt, 2013). Many facultative anaerobes rapidly become anaerobic as the oral biofilm develops, which explains the predominance of obligate anaerobic bacteria in the mouth (Wade, 2013).

Hyperbaric hyperoxia in the divers’ breathing gas is a likely contributing factor to the bacterial shift we observed. It has been noted that hyperoxia gave a selective relative growth advantage to oxygen-tolerant respiratory microbial species (e.g., *Staphylococcus aureus*) in patients with respiratory failure who received high concentrations of therapeutic oxygen. In parallel, using neonatal and adult mouse models, the same authors demonstrated that lung and gut bacterial communities were altered within 24 and 72 h, respectively, in mice exposed to hyperoxia (Ashley et al., 2020). To overcome the toxic effects of oxygen, both aerobic and facultative anaerobic organisms contain a highly regulated complex of antioxidant defense enzymes such as catalase or superoxide dismutase as well as other enzymatic and non-enzymatic defense mechanisms against the toxic effects of reactive oxygen species (ROS; Brioukhanov and Netrusov, 2007; Henry et al., 2014). These have been particularly well-described in the two pathogens in the species *Neisseria* (*Neisseria gonorrhoeae* and *Neisseria meningitidis*), when even within the same genus, Neisserial species appeared to have different contents of antioxidant enzymes (Archibald and Duong, 1986; Seib et al., 2004, 2006). Our divers were exposed to hyperoxia during the bottom phase, with ppO_2_ of 40 kPa in the pressurized living chambers and up to 60–80 kPa during the bell-run excursions, which is double to triple the amount of oxygen in normobaric air.

An increase in oxygen availability has been reported to induce gut dysbiosis, thus driving an uncontrolled luminal expansion of the family *Enterobacteriaceae*, which are facultative anaerobic (Rivera-Chavez et al., 2017). Nevertheless, it is difficult to draw comparisons between the oral and the gut microbiome in relation to the normoxic and hyperoxic conditions because the gut microbiome is usually more stable than the oral microbiome in terms of environmental conditions. However, we can compare the gut microbiome and the neonatal gut microbiome. The latter being more abundant in oxygen, it is frequently dominated by facultative anaerobes such as *Proteobacteria* species (Guaraldi and Salvatori, 2012). By consuming the oxygen in the 1st week of life, these facultative anaerobes create a more suitable environment for obligate anaerobes such as *Fusobacteria* (Kelly et al., 2018). The hyperoxic conditions experienced by the divers during the bottom phase may thus be inadequate for *Fusobacteria*, which might in turn leave space for *Proteobacteria*.

The gut microbiota also seems to be related to the host response to hypobaric hypoxia exposure associated with increased inflammation and risk of infection (Hartmann et al., 2000; Khanna et al., 2018). High altitude tends to be positively correlated with obligate anaerobes (Maity et al., 2013; Suzuki et al., 2019) and is associated to relative abundance of *Prevotella* (Karl et al., 2018). It has recently been described that intermittent hypoxia can induce alterations in the gut microbiota (Moreno-Indias et al., 2015; Ramos-Romero et al., 2020). Together, these results suggest that obligate anaerobes may have a competitive advantage under hypoxic conditions over aerobes that require oxygen.

What might the consequences be of a shift in diversity and abundance of bacteria during saturation diving? Most studies on the oral microbiome established associations between oral health conditions and the bacterial composition in saliva. Indeed, healthy periodontal conditions were mainly related to the genus *Neisseria*, while the predominance of the genera *Prevotella* and *Veillonella* was associated with periodontal diseases (Yamashita and Takeshita, 2017). In addition, many studies also associate the gut microbiome to human health. A raise of *Proteobacteria* abundance in the gut microbiome has been linked to pathologies such as obesity (Zhu et al., 2013) and Type 2 diabetes (Larsen et al., 2010; Zhang et al., 2013). *Proteobacteria* also appear to be associated with extraintestinal diseases such as asthma and chronic obstructive pulmonary disease (COPD), making it a common microbial signature of states linked to various degrees with inflammation (Rizzatti et al., 2017). However, it is still unclear whether a transient bacterial shift from *Fusobacteria* to *Proteobacteria* in the oral microbiota may have an impact on the divers’ health. Additional studies should be conducted to investigate the bacterial metabolic pathways altered during saturation diving.

## Limitations

This study imposed no restrictions on the divers’ routines or diet. Since they worked overlapping shifts over the 24-h day, the time for saliva collections varied accordingly. We can, therefore, not rule out possible effects of circadian variation which may have a functional impact on bacterial activity (Takayasu et al., 2017). The divers were also free to choose individually from daily selections of meals from the vessel galley. A modest association between diet and oral microbiota has been reported (Kato et al., 2017). However, these limitations are not expected to cause false-positive results.

## Conclusion

We identified changes in the abundance of three bacterial phyla in the oral microbiota during commercial heliox saturation diving: *Fusobacteria* decreased, whereas *Proteobacteria* and *Actinobacteria* increased during the bottom phase. At the genus level, there was a decrease in the relative abundance of *Fusobacterium*, *Leptotrichia*, *Oribacterium*, and *Veillonella*, and an increase of *Neisseria* and *Rothia*. However, no changes persisted at the end of the decompression. The transient nature of the change could imply that hyperbaric heliox saturation has no lasting effect on the oral microbiota, but it is unknown whether and how the bacterial shift during saturation may impact the divers’ health or well-being.

## Data Availability Statement

The datasets presented in this study can be found in online repositories. The names of the repository/repositories and accession number(s) can be found at: https://www.ebi.ac.uk/ena/browser/view/PRJEB40804.

## Ethics Statement

The studies involving human participants were reviewed and approved by Norwegian Regional Committee for Medical and Health Research Ethics (REK). The patients/participants provided their written informed consent to participate in this study.

## Author Contributions

RM, KH, and IE designed the study. IE collected the material. RM, JL, AH, and AF conducted the analyses. RM initiated the manuscript. All authors contributed in the writing and approval of the final version.

### Conflict of Interest

TechnipFMC sponsored helicopter transfers and boarding on the DSV Deep Arctic for IE.

The remaining authors declare that the research was conducted in the absence of any commercial or financial relationships that could be construed as a potential conflict of interest.

## Footnotes

^1^https://www.ebi.ac.uk/ena/browser/view/PRJEB40804
